# The safety and efficacy of mesenchymal stromal cells in ARDS: a meta-analysis of randomized controlled trials

**DOI:** 10.1186/s13054-022-04287-4

**Published:** 2023-01-20

**Authors:** Fengyun Wang, Yiming Li, Bingqing Wang, Jianguo Li, Zhiyong Peng

**Affiliations:** 1 Department of Critical Care Medicine, Zhongnan Hospital, Wuhan University, Wuhan, Hubei Province China; 2 Clinical Research Center of Hubei Critical Care Medicine, Wuhan, 430071 Hubei China; 3 Department of Critical Care Medicine, Center of Critical Care Nephrology, University of Pittsburgh School of Medicine, Pittsburgh, PA 15213 USA

**Keywords:** Mesenchymal stromal cells, Acute respiratory distress syndrome, Acute lung injury, Cell transplantation, Coronavirus disease 2019

## Abstract

Mesenchymal stromal cells (MSC) have shown potential efficacy in both animal and human trials of acute respiratory distress syndrome (ARDS). Especially during the COVID-19 pandemic, MSC was intensely studied for treating COVID-19-induced ARDS. The purpose of this study is to evaluate the safety and efficacy of MSC in ARDS via a meta-analysis of randomized controlled trials (RCTs). Therefore, a meta-analysis of RCTs of MSC as a therapy for ARDS was conducted. The protocol of this review was registered on Open Science Framework. With no language restriction and according to the “PICOs” principle, searches were conducted on Pubmed and Embase to retrieve any clinical literature on MSC for ARDS. Any RCT, which compared MSC to controls for ARDS, where MSC and controls were intravenously infused, of any dosage, was eligible for inclusion. A total of 13 RCTs, which evaluated MSC versus control for treating ARDS, enrolling a total of 655 cases, met the inclusion criteria and appeared in this meta-analysis. A heterogeneity assessment was carried out using the *χ*^2^ test, where a *P* value less than 0.05 was considered significant. The choice of a fixed-effect or a random-effect model was decided by the *I*^2^ value in each of the analyses. This meta-analysis indicated that there was no significant difference in terms of adverse events between MSC and control for ARDS (OR = 0.64, 95% CI [0.34, 1.20], *P* = 0.17, and *I*^2^ = 0%). In comparison with control, MSC could reduce the mortality of ARDS (OR = 0.66, 95% CI [0.46, 0.96], *P* = 0.03, and *I*^2^ = 10%). Based on the results of our meta-analysis, the safety of MSC was demonstrated to be non-inferior to that of standard treatment, and MSC may reduce the mortality rate of ARDS. Though the heterogeneity in the main results was low (*I*^2^ < 25%), more high-quality and large-scale clinical trials are needed to further confirm our findings.

**Supplementary Information:**

The online version contains supplementary material available at 10.1186/s13054-022-04287-4.

## Introduction

Acute respiratory distress syndrome (ARDS) is a life-threatening clinical syndrome with high morbidity and mortality, which is featured by acute non-cardiogenic lung edema, hypoxia refractory to routine oxygenation, and severe respiratory distress [1]. According to the “Lung Safe” international epidemiological investigation, the mortality of ARDS ranged from 34.9 to 46.1% and the prevalence of it accounted for 10.4% of all ICU admissions around the globe [2]. Though lung-protective ventilation [3], controlling driving pressure [4], prone position [5], and ECMO [6] were identified as effective measures, the mortality of ARDS was still unacceptably high. Apart from low-dose corticosteroids (such as 6 mg/day dexamethasone) and remdesivir were recommended for treating COVID-19-induced ARDS [7], there is no other guideline-recommended therapy directly targeting the pathophysiology of this lethal clinical syndrome.

Mesenchymal stromal cells (MSC) belonging to a member of pluripotent stem cells, are of stromal origin and can be extracted from bone marrow, adipose tissue, umbilical cord, etc. [8]. MSCs are considered candidates for the treatment of ARDS because they can be deployed to the injured sites, where they are shown to repair tissue through its paracrine and anti-fibrosis effects in animal models of ARDS induced by endotoxin [9]. Additionally, MSC may transfer mitochondria into alveolar epithelium to improve bioenergetics of lung tissue and improve lung function [10]. The secretome released by MSC also is demonstrated to possess anti-inflammatory effects and is protective in animal models of ARDS [11]. Through the release of lipocalin-2 and LL-37, MSC has been shown to possess antimicrobial effects, possibly by enhancing the phagocytic activity of host immune cells [12]. In addition, MSC has been reported to preserve the integrity of vascular endothelial and alveolar epithelial barrier in preclinical models of ARDS [13]. Beyond that, in lung injuries induced by endotoxin, MSC is able to improve alveolar fluid clearance [14]. By exhibiting multipotent characteristics such as tissue repair, regeneration, antimicrobial, and anti-inflammation, MSC was widely investigated in ARDS animal models and was considered as a promising therapy for ARDS [15].

In the last decade, clinical trials have been conducted to investigate the safety and efficacy of MSC concerning ARDS [16–23]. However, due to the small sample size of these early clinical trials, the potency of MSC for ARDS is still subject to question and thus merits further discussion and investigation. Toward this end, we conducted a meta-analysis of randomized controlled trials of MSC in patients with ARDS to review the safety and efficacy of MSC for ARDS. The main outcomes of this meta-analysis were treatment-related adverse events (AEs) and all-cause mortality.

## Materials and methods

### Data sources

The protocol of this review was registered on Open Science Framework (OSF), registration https://doi.org/10.17605/OSF.IO/V74XA. PubMed and EMBASE (up to November 2022) were searched to identify relevant clinical trials with a tailored search strategy. Trials other than randomized controlled trials (RCT) were excluded from further screening. Search terms included ‘Mesenchymal Stromal Cells,’ ‘Mesenchymal Stem Cells’ ‘MSC,’ ‘Acute Respiratory Distress Syndrome,’ ‘ARDS,’ ‘Acute Lung Injury,’ and ‘ALI,’ and they were combined by patients, intervention, control, and outcomes (PICOs) principle. No language restriction was set in the database search. The search strategy is as follows: (((((Acute Respiratory Distress Syndrome[Title/Abstract]) OR (ARDS[Title/Abstract])) OR (acute lung injury[Title/Abstract])) OR (ALI[Title/Abstract])) AND ((((Mesenchymal Stem Cells[Title/Abstract]) OR (Mesenchymal Stromal Cells[Title/Abstract])) OR (MSC[Title/Abstract])) OR (MSCs[Title/Abstract]))) AND ((((((((control[Title/Abstract]) OR (randomized[Title/Abstract])) OR (randomly[Title/Abstract])) OR (controlled[Title/Abstract])) OR (RCT[Title/Abstract])) OR (placebo[Title/Abstract])) OR (sham[Title/Abstract])) OR (random[Title/Abstract])).

### Study selection

Two authors (FYW and YML) independently searched and scrutinized literature on databases and read the title and abstract of each retrieved article to determine which of them required further assessment. Full texts of articles were retrieved when the information given in the titles and abstracts indicated that the study adopted a prospective design to compare MSC with control in patients with ARDS. When disputes existed, they were discussed thoroughly to reach a consensus. The inclusion criteria were (1) any RCTs that compared MSC with controls for ARDS, (2) included patients who were adults, of any gender, and had an established ARDS, (3) MSC intravenously infused, of any dosage; and controls or placebo intravenously infused, of any dosage.

### Data extraction

Review authors (FYW and YML) independently extracted data with a customized data extraction form. The data extraction form included the following detailed information: (1) year of publication, (2) the number of included patients, (3) descriptions of dose, route, and timing of MSC and controls, (4) treatment-related AEs, all-cause mortality and other secondary outcomes.

### Analyzed outcomes

The primary outcomes of this review were treatment-related AEs and all-cause mortality at 28 days. The secondary outcomes included clinical data such as ICU length of stay, PiO_2_/FiO_2_; and inflammatory biomarkers such as IL-6 and IL-8.

### Data analysis and statistical methods

Data analyses of this review were performed by the Review Manager (Version: 5.4, Cochrane Collaboration, UK). Clinical heterogeneity was assessed in the population, methodology, and in interventions and outcomes of each study to assess whether the pooling of results was feasible. Values of *I*^2^ less than 25% were considered low in heterogeneity, for which the fixed-effect model of meta-analysis was used, whereas values of *I*^2^ between 25 and 75% were considered moderate in heterogeneity and a random-effects model was used. Values of *I*^2^ higher than 75% indicated high levels of heterogeneity, in which case no meta-analysis was performed. All statistical tests were two-sided and a *P* value less than 0.05 was considered statistically significant. Dichotomous variables such as treatment-related AEs and all-cause mortality expressed in ratios were extracted. Continuous variables such as inflammatory biomarkers IL-6 and IL-8 expressed in mean and standard deviation were extracted. Serum IL-6 and IL-8 examined 5 days or 7 days after trial drug or placebo administration were to be extracted in our review.

### Heterogeneity exploration and quality assessment

A heterogeneity assessment was performed using the *χ*^2^ test, where a *P* value less than 0.1 was considered as the significance set. The funnel plot was utilized to detect any possible publication bias. The quality of the included literature was assessed by the Cochrane Collaboration tool for assessing risk of bias, which contains the following five aspects: sequence generation, allocation concealment, blinding, incomplete outcome data, and selective outcome reporting. The assessment of risk of bias was presented by using a “risk of bias summary figure,” which presents all of the judgments in a cross-tabulation of study by entry. This display of internal validity indicates the weight the writer may give to the results of each study.

## Results

### Study selection process

**Fig. 1:**
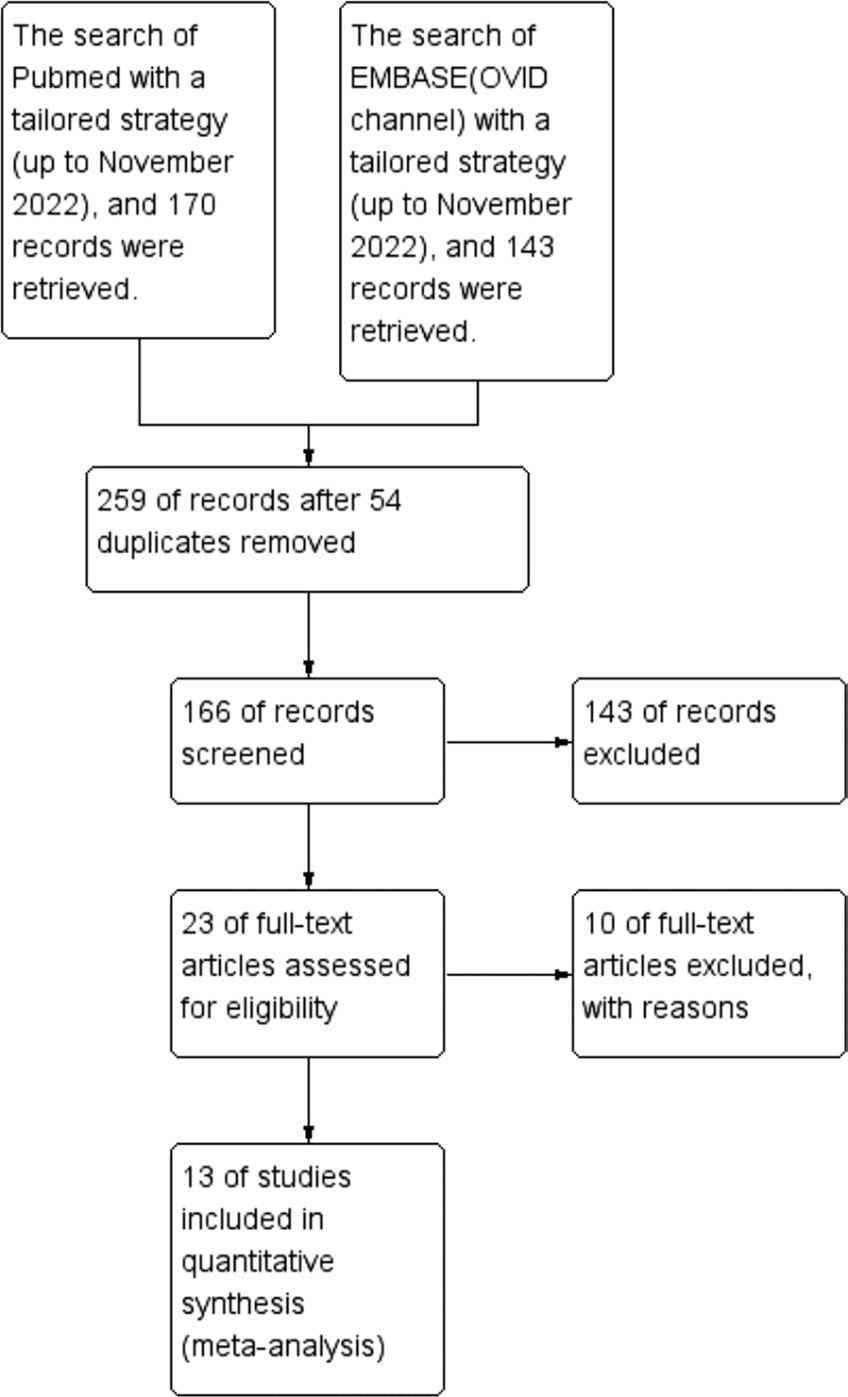
The flow diagram of the literature search process

The whole search and selection process of the electronic databases was shown in the flow diagram (Fig. 1). Specifically, 170 articles were retrieved from Pubmed and 143 articles were retrieved from Embase. After duplicates were removed, a total of 259 articles were retrieved. After reading the titles and abstracts of each of the retrieved articles, the 166 retrieved articles were preserved and the full text of 23 of them was obtained for further examination. Seven papers were eliminated from consideration because they were either case series [23] or uncontrolled safety studies [22] or a study protocol [24–28]. Another three papers [29–31] were discarded because they reported the same trials as the included studies [17, 18, 32] did. These three excluded studies were only the secondary analysis of the three relevant studies included in our review and they didn’t report outcomes analyzed in our study. Finally, 13 papers met the inclusion criteria and were included in this meta-analysis [16–19, 32–40]. MSC or controls were initiated once the patients met the Berlin definition of ARDS or severe/critical COVID-19 in all the included studies after randomization.

### Characteristics of the included studies

The main characteristics of the 13 studies including the type of study design, patients’ characteristics, dose and treatment duration of the studied medicine, population, and outcomes are presented in Table 1. The etiology of ARDS was not restricted to one specific disease in two included studies [16, 17], whereas, in the other 11 studies, ARDS was solely induced by COVID-19. The average age of the patients in the included studies ranged from 53 to 69.8 years old, and in terms of which, there was no significant difference between the MSC group and the Control group (*P* = 0.55, Additional file 1: Fig. S1A). Male patients accounted for 66.1% of the MSC group and 66.3% of the Control group (*P* = 0.77, Additional file 1: Fig. S1B). MSC was only used in patients with moderate-to-severe ARDS in six included studies, but in the other seven studies, either the severity was not defined, or MSC can be used for all patients with ARDS, regardless of the severity of the disease. Four included studies held a modality of multi-center RCT [17, 19, 34, 38], while the other nine studies were just one single-center RCTs. The method of randomization and allocation concealment was not thoroughly elucidated in four included trials [16, 18, 19, 37]. The source origins of the MSCs included adipose, bone marrow, umbilical cord, etc., and the dose of MSCs ranged from 1 × 10^6^ to 100 ± 20 × 10^6^ in included studies.

**Table 1:** The main characteristics of the studies included in the meta-analysis

Reference	Design	Age (MSC vs. Control, years)	Gender (male ratio)	Population	Group(dose, treatment duration)		ITT Population	Outcomes
					MSC	Control		
Guoping Zheng [16]	RCT	66.7 ± 20.4 vs. 69.8 ± 9.1	6/6 vs. 5/6	N = 12 (1:1), adult patients who met the Berlin definition of moderate and severe ARDS	AD MSCs, 1 × 10^6^ cells/kg of body weight, one IV dose	NS	12 (6 vs. 6)	Adverse events Oxygenation index, length of hospital stay, ventilator-free days and ICU-free days at day 28, and SP-D, IL-6 or IL-8 levels in serum
Michael Matthay [17]	RCT	55 ± 17 vs. 55 ± 20	23/40 vs. 10/20	N = 60 (2:1), ventilated adult patients with moderate-to-severe ARDS	BM-MSCs, 10 × 10^6^ MSC/kg (pbw), one IV dose	Placebo	60 (40 vs. 20)	Infusion-associated adverse events All-cause mortality, ventilator-free days, intensive-care-free days, days free from organ failure, SOFA score, oxygenation index, and lung injury score Angiopoietin 2, RAGE, IL-6, or IL-8 levels in serum
Giacomo Lanzoni [18]	RCT	58.58 ± 15.93 vs. 58.83 ± 11.61	5/12 vs. 8/12	N = 24 (1:1), adult patients hospitalized for severe COVID-19	UC-MSCs, 100 ± 20 × 10^6^ UC-MSCs, 2 IV doses	50 mL vehicle solution	24 (12 vs. 12)	Infusion-associated adverse events, cardiac arrest or death within 24 h post-infusion, and incidence of AEs. Survival, time to recovery, and AEs within 31 days
Ismail Dilogo [19]	RCT	–	15/20 vs. 15/20	N = 40 (1:1), intubated patients with severe COVID-19 (moderate and severe ARDS)	UC-MSCs, 1 × 10^6^ cells/kg body weight, one IV dose	100 ml NS	40 (20 vs. 20)	Mortality rate, length of ventilation, Length of ICU stay, AE or serious AE (SAE) Routine blood count, differential count, CRP, D-dimer, fibrinogen, and procalcitonin, VEGF, ferritin, IL-6, LIF, CX-CR3 in serum
G Adas [33]	RCT	–	–	N = 20 (1:1), COVID-19 patients with severe ARDS	WJ MSCs, 3 × 10^6^ cells/kg body weight, three IV doses	Placebo	20 (10 vs. 10)	Adverse events, mortality, lymphocyte subgroups, and inflammation markers such as CRP, PCT, and Ferritin
Antoine Monsel [34]	RCT	64 ± 10.4 vs. 63.2 ± 11.4	17/21 vs. 20/24	N = 45, COVID-19 patients with ARDS	UC-MSCs, 3 × 10^6^ cells/kg body weight, IV	150 ml NS	45 (21 vs. 24)	Respiratory improvement, SOFA scores, PaO2/FiO2 ratios, ventilation-free days and 28-day mortality; adverse events; and inflammatory biomarkers
Carmen Rebelatto [35]	RCT	53 ± 15.3 vs. 61.7 ± 9.7	8/11 vs. 4/6	N = 17, COVID-19 patients with moderate/severe ARDS	UC-MSCs, 5 × 10^6^ cells/kg body weight, IV	Placebo	17 (11 vs. 6)	Adverse events, mortality, PaO_2_/FiO_2_; ferritin, IL-6, CRP, D-dimer, and neutrophils
Hamid Aghayan [36]	RCT	62.3 vs. 58.4	6/10 vs. 8/10	N = 20 (1:1), COVID-19 patients with ARDS	PL-MSCs, 1 × 10^6^ cells/kg body weight, IV	Placebo	20 (10 vs. 10)	Adverse events, mortality, and lymphocyte subgroups
Lei Shi [32]	RCT	60.72 ± 9.14 vs. 59.94 ± 7.79	37/65 vs. 19/35	N = 100, COVID-19 patients with ARDS	UC-MSCs, 4 × 10^6^ cells/kg body weight, IV	Placebo	100 (65 vs. 35)	The proportion of whole lung lesion volumes, adverse events, and mortality
Lei Shu [37]	RCT	61.00 ± 17.87 vs. 57.86 ± 15.79	8/12 vs. 16/29	N = 41, COVID-19 patients with ARDS	UC-MSCs, 2 × 10^6^ cells/kg body weight, IV	100 ml NS	41 (12 vs. 29)	Mortality, the time to clinical improvement, adverse events, and lab indexes
Michael Bowdish [38]	RCT	61.8 ± 13.0 vs. 59.6 ± 13.8	79/112 vs. 75/110	N = 222, COVID-19 patients with moderate/severe ARDS	BM-MSCs, 2 × 10^6^ cells/kg body weight, IV	Placebo	222 (112 vs. 110)	Mortality, mechanical ventilation days, clinical improvement, ICU length of stay, and adverse events
Najmeh Farkhad [39]	RCT	–	7/10 vs. 6/10	N = 20, COVID-19 patients with mild/moderate ARDS	UC-MSCs, 1 × 10^6^ cells/kg body weight, IV	Placebo	20 (10 vs. 10)	Mortality, PaO_2_/FiO_2_, lung imaging, and inflammatory biomarkers such as IL-1 beta, IL-6, and TNF-a
Xiaowei Xu [40]	RCT	58.31 ± 12.49 vs. 61.11 ± 11.03	17/26 vs. 13/18	N = 44, COVID-19 patients with ARDS	UC-MSCs, 9 × 10^6^ cells/kg body weight, IV	Placebo	44 (26 vs. 18)	Mortality, clinical improvement, PaO_2_/FiO_2_, inflammatory indices (including CRP and IL-6), and adverse events

*ITT* Intention to treat; *AD MSCs* Adipose-derived MSCs; *BM-MSCs* Bone Marrow MSCs; *UC-MSCs* Umbilical cord MSCs; *IV* Intravenous; *SOFA* Sequential Organ Failure Assessment; *NS* Normal saline; *COVID-19* Coronavirus disease 2019

### The meta-analysis of the primary outcomes

**Fig. 2:**
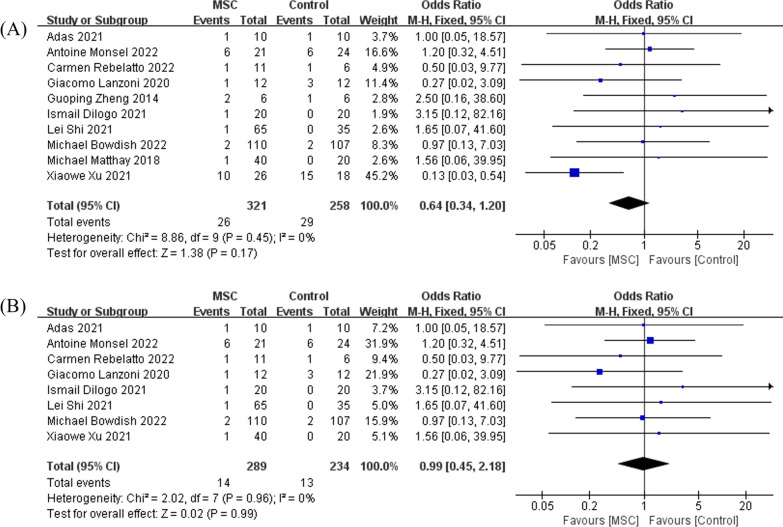
The meta-analyses of adverse events, comparing MSC with the control: **A** the comparison of MSC with control in general ARDS; **B** the comparison of MSC with control in COVID-19-induced ARDS. The size of each square represents the proportion of information given by each trial. Crossing with the vertical line suggests no difference between the two groups

Regarding treatment-related AEs, the pooling results of 10 RCTs, enrolling a total of 579 patients, suggested that in comparison with control, MSC infusion did not increase any pre-defined AEs in treating ARDS (OR = 0.64, 95% CI [0.34, 1.20], *P* = 0.17, and *I*^2^ = 0%), Fig. 2A. For the COVID-19-induced ARDS subgroup, the pooled results of eight RCTs indicated that when compared with control, MSC did not increase any treatment-related AEs (OR = 0.99, 95% CI [0.45, 2.18], *P* = 0.99, and *I*^2^ = 0%), Fig. 2B. When the random-effects model was adopted, the results remained unchanged (Additional file 1: Fig. S2A and B).

**Fig. 3:**
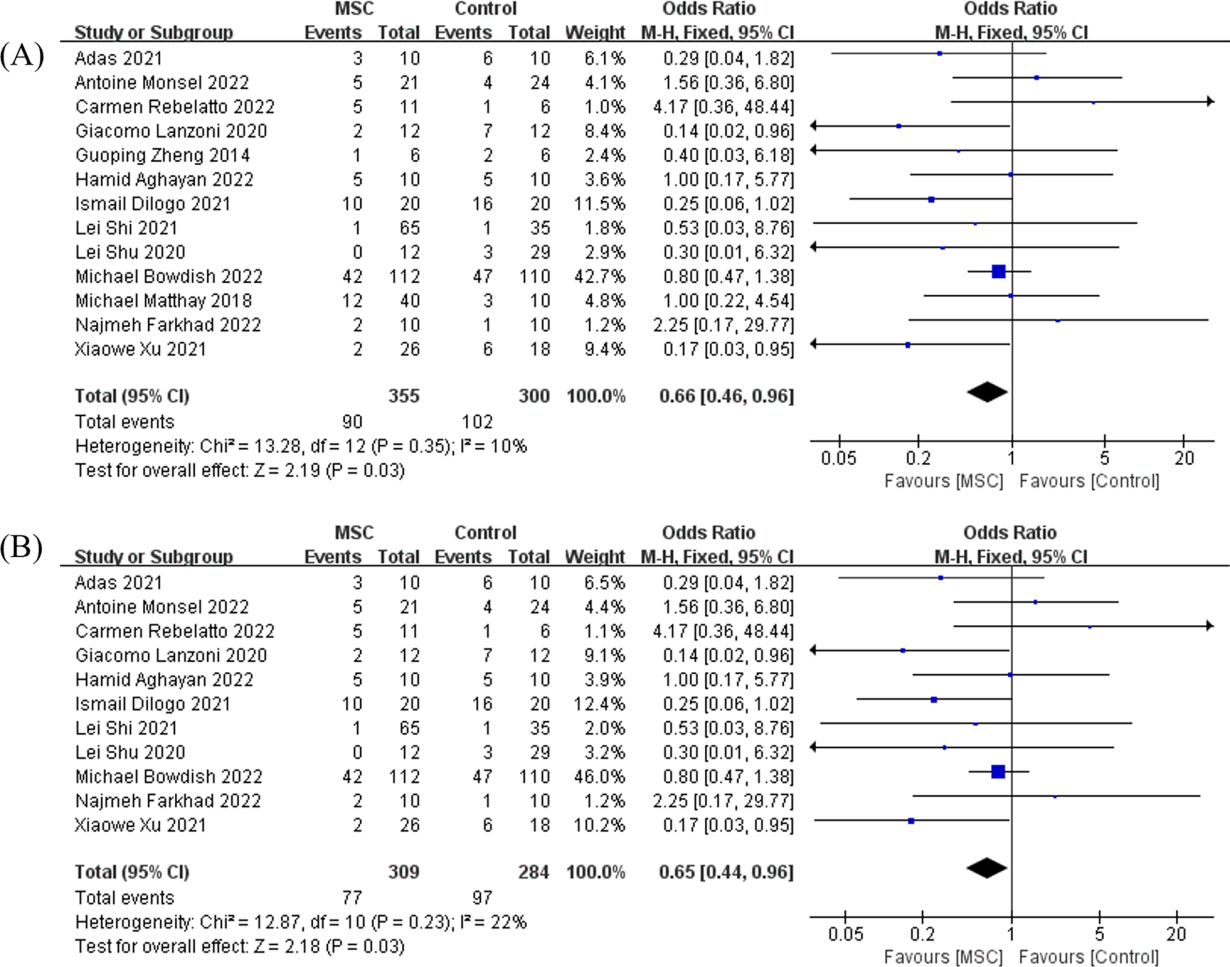
The meta-analyses of mortality, comparing MSC with the control: **A** the comparison of MSC with control in general ARDS; **B** the comparison of MSC with control in COVID-19-induced ARDS. The size of each square represents the proportion of information given by each trial. Crossing with the vertical line suggests no difference between the two groups

As for 28 days all-cause mortality, 13 studies with a total of 655 patients enrolled, the synthesized data indicated that compared with control, MSC reduced the mortality rate in adult patients with ARDS (OR = 0.66, 95% CI [0.46, 0.96], *P* = 0.03, and *I*^2^ = 10%), Fig. 3A. When the model of meta-analysis was adjusted to a random-effects model, the difference remained significant and the *P* value was 0.05 (Additional file 1: Fig. S3A). For the COVID-19-induced ARDS subgroup, 11 studies with a total of 593 patients were included, and the pooled results proved that compared with controls, MSC reduced mortality in COVID-19 patients with ARDS (OR = 0.65, 95% CI [0.44, 0.96], *P* = 0.03, and *I*^2^ = 22%), Fig. 3B. Of note, when the random-effects model was adopted, the *P* value was 0.07 (Additional file 1: Fig. S3B).

**Fig. 4:**
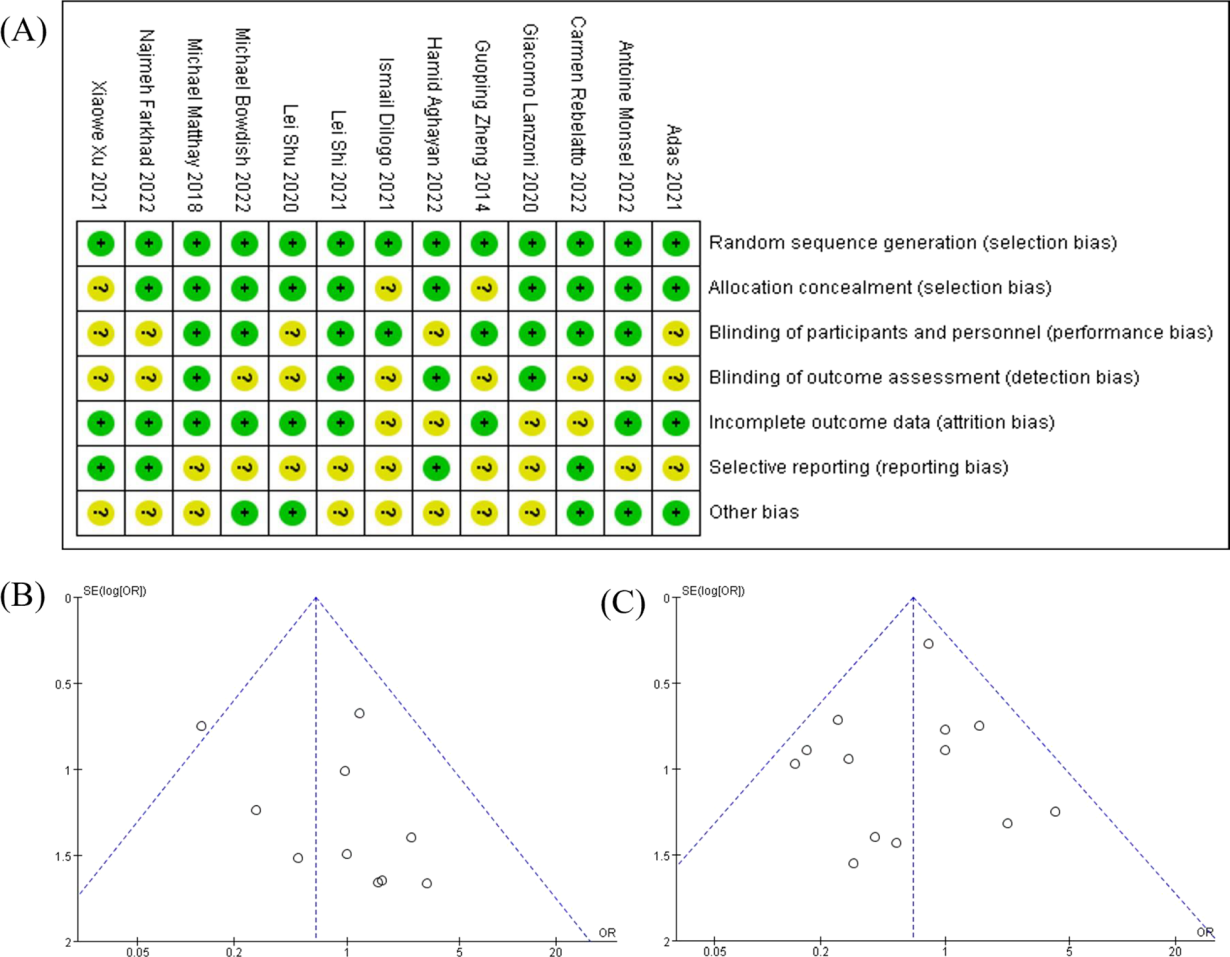
The assessment of possible bias: **A** The risk of bias summary graph: review authors' judgements about each risk of bias item for each included study. **B** The funnel plot for adverse events. **C** The funnel plot for mortality

The risk of bias summary for the included trials is presented in Fig. 4A. The general heterogeneity is low among these studies, and therefore, it is possible to pool them for meta-analyses. The funnel plot is utilized to detect any possible publication bias. As expressed in Fig. 4B, C the majority of the studies included in the meta-analyses are distributed symmetrically. Therefore, the publication bias in the present analysis is low and acceptable.

### The summarization of secondary outcomes

The meta-analysis of secondary outcomes was not conducted either because the data were not extractable or not presented. Six included studies reported the effect of MSC on oxygenation. Though three included studies implied that MSC may increase PaO_2_/FiO_2_ ratio [16, 37, 39], the other three studies suggested that MSC did not have much impact on PaO_2_/FiO_2_ ratio at any timepoints [17, 19, 34]. On ventilation-free days to 28 days in ARDS, five studies didn’t detect any significant difference between MSC and controls [16, 17, 19, 34, 38]. In terms of ICU-free days, although five studies discovered no significant difference between the two groups [16, 19, 34, 38, 40], one study revealed that MSC may reduce ICU-free days in ARDS [17]. Meanwhile, the effects of MSC on serum IL-6 in ARDS were also controversial, as while three studies suggested no significant difference detected [16, 17, 40], four others implied that MSC may downregulate serum IL-6 [18, 19, 35, 39]. Additionally, three included studies reported no significant impact of MSC on serum IL-8 in ARDS [16, 17, 35].

## Discussion

Our meta-analysis summarized the results of currently available RCT studies focused on MSC for ARDS and determined that the safety of MSC was not inferior to that of standard treatment. Second, with the treatment of MSC, the short-term survival of ARDS was improved. Third, the impact of MSC on oxygenation, ventilation-free days, ICU-free days, and systemic inflammation was still inconclusive thus far because no meta-analysis was done for these important outcomes.

No discrepancy regarding treatment-related adverse events was observed between MSC and controls in the 10 included RCTs, indicating the safety of MSC is reliable and further studies are warranted. In the COVID-19-induced ARDS subgroup analysis, of AEs, there are still no significant differences between MSC and control. Thus, MSC is safe for treating severe COVID-19. Since our meta-analyses showed that mortality is reduced in both general ARDS and COVID-19-induced ARDS, MSC can be further investigated as a promising therapy for ARDS. Though *I*^2^ < 25%, when the random-effects model of meta-analysis was used, the *P* value of the subgroup analysis of COVID-19-induced ARDS exceeded 0.05 (*P* = 0.07). Although the subtle difference in random-effects model would not undermine the findings of mortality, more MSC studies are needed to consolidate its protective effect in COVID-19-induced ARDS. In our meta-analysis, albeit improved survival with the treatment of MSC, three included studies indicated that compared with control, oxygenation was not improved, this may suggest that the improvement of survival by MSC was not primarily dependent on oxygenation for its effectiveness. The paracrine of growth factors, promotion of tissue repair and regeneration, and the anti-inflammatory effects of MSC [41, 42] may comprehensively alter the pathophysiological progress of ARDS. However, the particular mechanism awaits future studies to decode.

Regarding secondary outcomes, because of the different modalities used in data presentation, not enough data can be extracted. For this reason, no meta-analysis was conducted for secondary outcomes. Of note, despite no difference reported in the incidence of AEs and ventilation-free days, the study by Michael Matthay et al. [17] revealed that ICU-free days were reduced in the MSC group. They also detected nonsignificant elevated mortality with the treatment of MSC for ARDS (12/40 in the MSC and 3/20 in the control died). However, they acknowledged that mortality, as expected, was higher in the group of MSC than in the control group and that this was due to higher severity of the disease in the first group than in the latter group [17].

So far, due to a lack of effective targeted treatments, ARDS is still one of the most deadly clinical syndromes in the critical care field even after more than half a century of its discovery [43, 44]. Even for patients who survived this purgatory, their quality of life inevitably and dramatically declined because of their substantially damaged and not fully recovered lung function [45]. Especially after COVID-19 had swept all over the globe in the last three years and caused millions of deaths [46, 47], effective and available therapies for ARDS are quite needed.

In the last decade, cell therapy including MSC has been clinically investigated in a variety of pulmonary diseases. In 2013, Daniel Weiss et al. investigated the safety and efficacy of MSC in COPD. Though they didn’t observe any significant differences in pulmonary function or life-quality indicators, the safety of MSC was found to be satisfying and an anti-inflammatory effect of MSC was detected as it can decrease circulating CRP [48]. For preterm infants with bronchopulmonary dysplasia, intratracheal transplantation of allogeneic UC-MSC was also found to be safe and feasible [49, 50]. In the phase 1 clinical trial conducted by Jennifer Wilson et al., the dose-escalation of MSC from 1 × 10^6^ to 10 × 10^6^ MSC/kg was well tolerated by patients with moderate-to-severe ARDS, and no infusion-associated AEs and serious AEs were observed during the trial [22]. A compassionate treatment trial of COVID-19-induced ARDS with UC-MSC was demonstrated to be safe, yet the improvement of oxygenation may have been attributable to the effects of MSC or the evolution of the course of the disease itself. This needs to be validated by more controlled trials [23]. Furthermore, not only was MSC clinically investigated for treating ARDS but MSC-derived therapies such as exosomes of MSC were also considered for treating this syndrome [11]. In a cohort study, BM-MSC-derived exosomes were demonstrated to be safe and could restore oxygenation and downregulate cytokines for the treatment of severe COVID-19 [51].

Though MSC may be a promising therapy for ARDS, how to use it correctly in ARDS is still an issue that many clinicians are concerned about. According to the summary of the dosage of MSC in our study, one dose or several doses of 1 × 10^6^ cells/kg of MSC seems to be safe in ARDS since this dosage didn’t increase any treatment-related AEs. Umbilical cord (UC) MSC was used in 8 of the 13 included studies, and given its high availability, it may be one of the most promising MSCs in the area of ARDS. Diana Islam et al. discovered that the effect of MSC in ARDS was determined by the microenvironment at the time of administration [52]. They proved that MSC might worsen ARDS in a microenvironment of high levels of IL-6 and fibronectin along with low antioxidant capacity. Correcting this adverse microenvironment with anti-oxidants or anti-inflammatory factors can reverse the detrimental effects of MSC. The aforementioned findings might guide us to use MSC in ARDS correctly. A combination of MSC with anti-oxidants and anti-inflammatory factors may be more beneficial for the treatment of ARDS.

There are several limitations within our meta-analysis. First, the sample size is small because the clinical investigation of MSC in ARDS is still at an early stage. Second, not enough data on secondary outcomes were extracted and no related meta-analysis was conducted. Third, because 11 of the 13 included studies were focused on COVID-19-induced ARDS, the evidence for non-COVID-19 ARDS is still scarce. Finally, male patients constituted about 66% of the total population, leading to the imbalance of the female-to-male ratio, which might be a source of clinical heterogeneity and limit the interpretation of the effects of MSC on female patients.

## Conclusion

Though 13 studies were included, the sample size (655 cases) was small. According to the results of our meta-analysis, the administration of MSC in adult patients with ARDS tended to be safe and feasible, and that MSC may possess the potential to improve the survival of ARDS. However, more high-quality, well-designed studies aiming to engineer and explore the beneficiary effects of MSC in ARDS are necessary and expected.

## Supplementary Information

**Additional file 1: Fig. S1.** The meta-analyses of age and gender (male patients ratio), comparing MSC with the control: **A** the comparison of MSC with control regarding age; **B** the comparison of MSC with control regarding gender. The size of each square represents the proportion of information given by each trial. Crossing with the vertical line suggests no difference between the two groups. **Fig. S2.** The meta-analyses of adverse events, comparing MSC with the control in the random-effects model: **A** the comparison of MSC with control in general ARDS; **B** the comparison of MSC with control in COVID-19-induced ARDS. The size of each square represents the proportion of information given by each trial. Crossing with the vertical line suggests no difference between the two groups. **Fig. S3.** The meta-analyses of mortality, comparing MSC with the control in the random-effects model: **A** the comparison of MSC with control in general ARDS; **B** the comparison of MSC with control in COVID-19-induced ARDS. The size of each square represents the proportion of information given by each trial. Crossing with the vertical line suggests no difference between the two groups

## Data Availability

Data sharing does not apply to this article as no new data were created or analyzed in this study.

## Abbreviations

ARDS: Acute respiratory distress syndrome
ALI: Acute lung injury
BM: Bone marrow
UC: Umbilical cord
AD: Adipose-derived
MSCs: Mesenchymal stem cells
