# Supplementary material for: The safety and efficacy of mesenchymal stromal cells in ARDS: a meta-analysis of randomized controlled trials
Source: Crit Care. 2023 Jan 20;27:31. doi: 10.1186/s13054-022-04287-4 (PMC9857915; doi:10.1186/s13054-022-04287-4)
Supplement: Supplementary file 1 — Additional file 1: Fig. S1. The meta-analyses of age and gender (male patients ratio), comparing MSC with the control: A the comparison of MSC with control regarding age; B the comparison of MSC with control regarding gender. The size of each square represents the proportion of information given by each trial. Crossing with the vertical line suggests no difference between the two groups. Fig. S2. The meta-analyses of adverse events, comparing MSC with the control in the random-effects model: A the comparison of MSC with control in general ARDS; B the comparison of MSC with control in COVID-19-induced ARDS. The size of each square represents the proportion of information given by each trial. Crossing with the vertical line suggests no difference between the two groups. Fig. S3. The meta-analyses of mortality, comparing MSC with the control in the random-effects model: A the comparison of MSC with control in general ARDS; B the comparison of MSC with control in COVID-19-induced ARDS. The size of each square represents the proportion of information given by each trial. Crossing with the vertical line suggests no difference between the two groups [file 13054_2022_4287_MOESM1_ESM.docx]

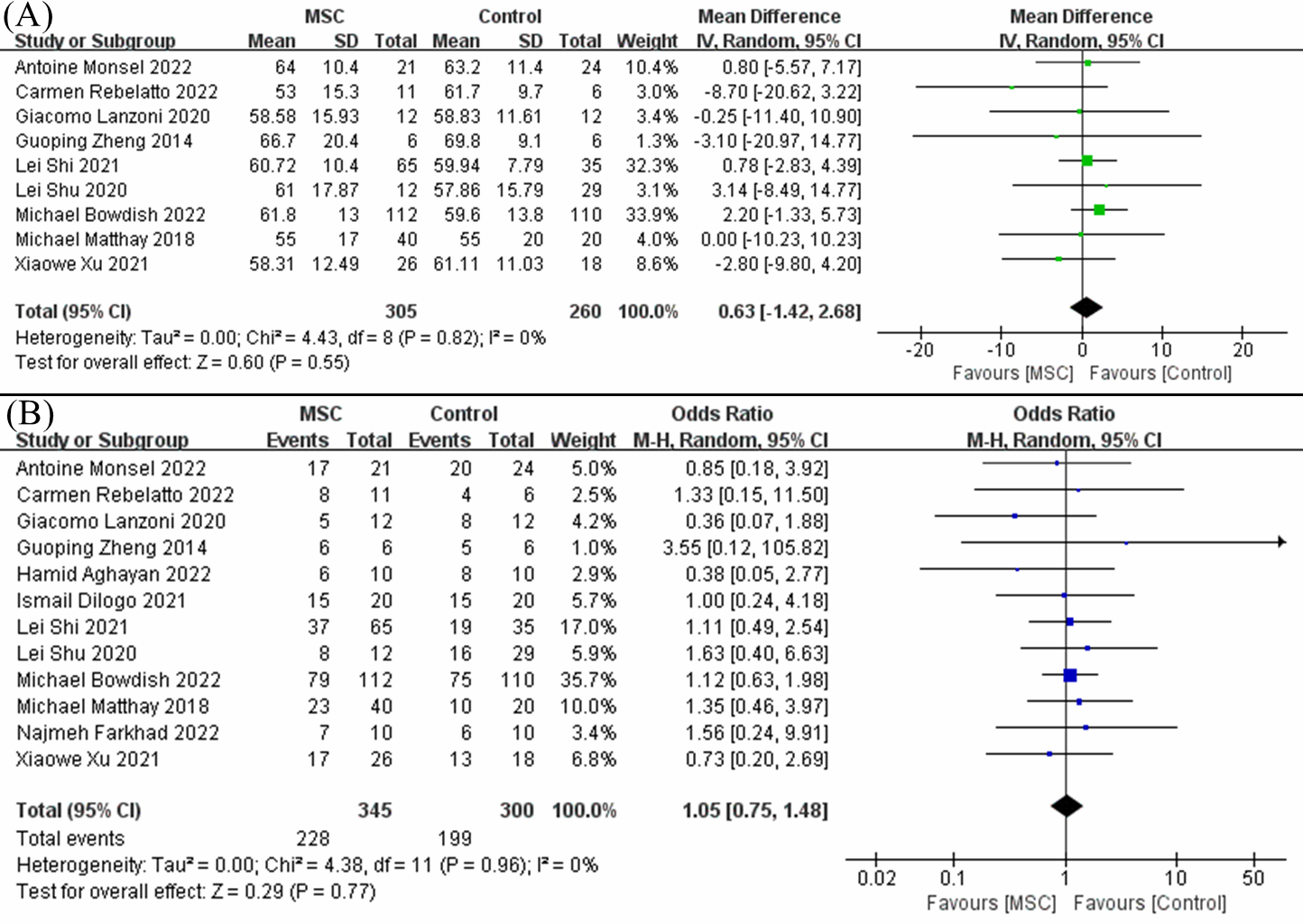


Fig.1S: The meta-analyses of age and gender (male patients ratio), comparing MSC with the control: (A) the comparison of MSC with control regarding age; (B) the comparison of MSC with control regarding gender. The size of each square represents the proportion of information given by each trial. Crossing with the vertical line suggests no difference between the two groups.


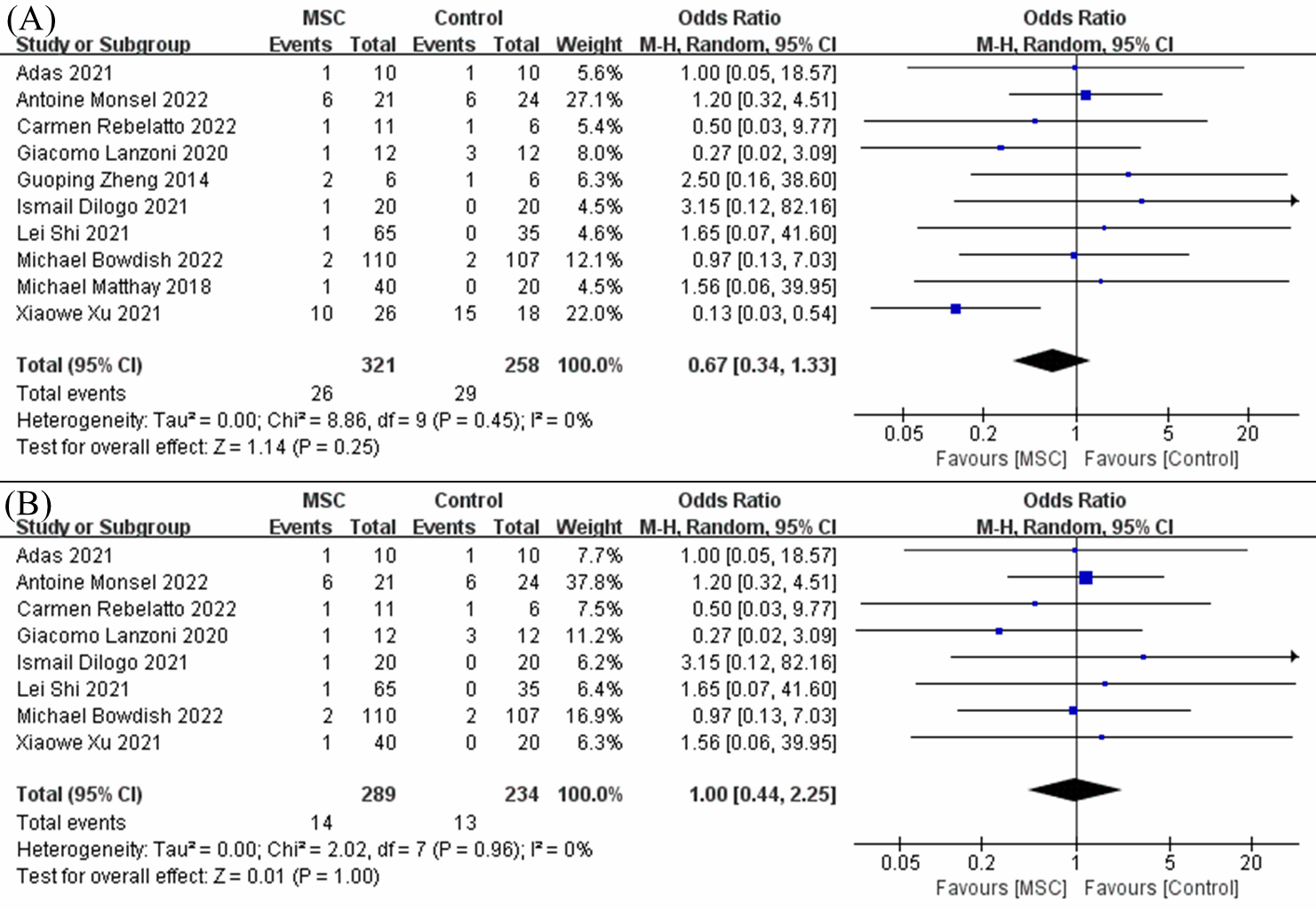


Fig.2S: The meta-analyses of adverse events, comparing MSC with the control in the random-effects model: (A) the comparison of MSC with control in general ARDS; (B) the comparison of MSC with control in COVID-19-induced ARDS. The size of each square represents the proportion of information given by each trial. Crossing with the vertical line suggests no difference between the two groups.


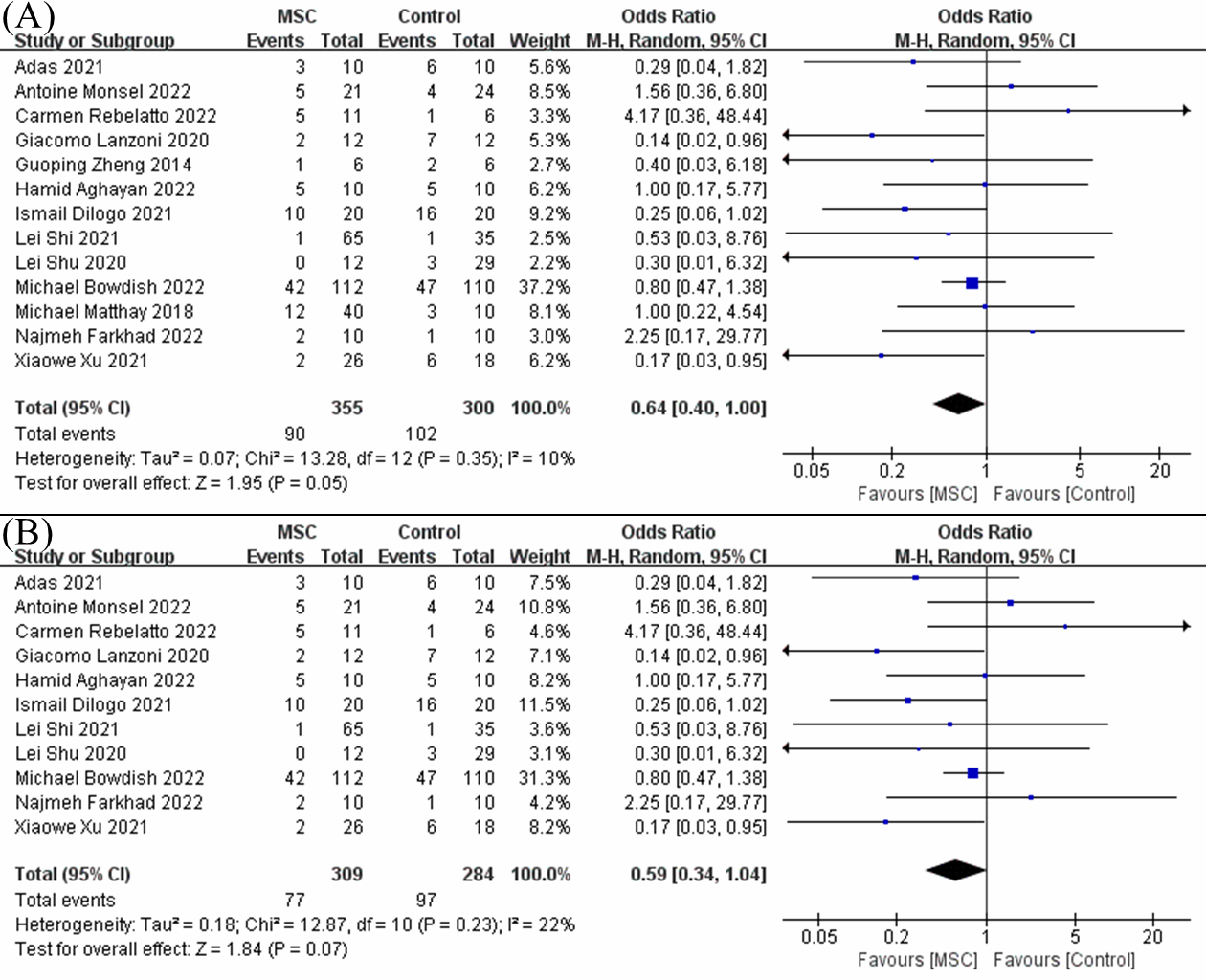


Fig.3S: The meta-analyses of mortality, comparing MSC with the control in the random-effects model: (A) the comparison of MSC with control in general ARDS; (B) the comparison of MSC with control in COVID-19-induced ARDS. The size of each square represents the proportion of information given by each trial. Crossing with the vertical line suggests no difference between the two groups.
